# A novel quantitation approach for maximizing detectable targets for offensive/volatile odorants with diverse functional groups by thermal desorption-gas chromatography-mass spectrometry

**DOI:** 10.1038/srep29248

**Published:** 2016-07-11

**Authors:** Yong-Hyun Kim, Ki-Hyun Kim

**Affiliations:** 1 Department of Civil and Environmental Engineering, Hanyang University, 222 Wangsimni-Ro, Seoul 04763, Korea

## Abstract

A multitude of analytical systems are needed to analyze diverse odorants with various functionalities. In this study, an experimental method was developed to assess the maximum covering range of odorants using a single experimental setup consisting of a thermal desorber-gas chromatography-mass spectrometry system. To this end, a total of 20 offensive odorants (aldehyde, ketone, ester, alcohol, aromatic, sulfide, amine, and carboxyl) were selected and tested by a single system. The analytical results of standards and environmental samples were evaluated in a number of respects. In the analysis of the standards, all targets were quantified via Carbopack (C + B + X) tube sampling while operating the thermal desorber at −25 °C. The method detection limits of 18 targets (exception of 2 out of the 20 targets: acetaldehyde and methanethiol) were excellent (mean 0.04 ± 0.03 ppb) in terms of their odor threshold values (74.7 ± 140 ~ 624 ± 1,729 ppb). The analysis of organic fertilizer plant samples at a pig farm (slurry treatment facility, compost facility, and ambient air) confirmed the presence of 18 odorants from 0.03 ppb (dimethyldisulfide, ambient sample) to 522 ppb (methyl ethyl ketone, slurry treatment facility). As such, our method allowed simultaneous quantitation of most key odorants with sufficient reliability and sensitivity.

There are many types of odorous compounds in air. The types and intensities of smells emitted by odorous substances are determined by their molecular weight, structure, and many other variables^1^,^2^. Following the example of other countries who have put effort into regulating the emission of malodorous substances, Korea established the offensive odor control law in 2004; this law lists a total of 22 odorous substances as key targets for regulation^3^.

In order to effectively control the emissions of odorous substances released into the air, proper experimental methods should be established for their quantitative analysis. In Korea, diverse analytical methods have been developed and recommended as the standard protocols to quantify and list of up to 22 key offensive odorants^3^,^4^. It is important to select the proper analytical method for specific odorant types because their detection should fit into a specific analytical method to appropriately balance different conditions (instrumentation) and properties (target compounds) such as the molecular structure, weight, and functional groups. For example, volatile fatty acids (VFA) with strong physical adsorptivity can undergo significant sorptive loss on the surface of the media used for their collection or storage. Thus, the sorbent tube (ST) method is recommended to reduce sorptive losses relative to grab-sampling approaches^5^. Additionally, in the case of sulfur or nitrogen compounds, specific detectors that are best-suited to detect each target should be used to gain maximum reliability^6^,^7^.

The Korea Ministry of Environment (KMOE) recommends the use of more than six different analytical methods for the analysis of the 22 designated odorants (Fig. 1). For instance, trimethylamine (TMA) is brought into contact with an acid solution (or acid-immersed filter) to initially induce the absorption of gaseous TMA. TMA is then extracted by solid phase microextraction (SPME), separated by gas chromatography (GC), and detected by a flame ionization detector (FID) or a nitrogen phosphorus detector (NPD). The analysis of TMA and many other designated odorants (i.e., reduced sulfur compounds (RSCs), aldehydes, and VFA) generally requires complicated pretreatment procedures for each instrumental setup for quantitative analysis. Thus, if one intends to conduct quantitative analysis of all the designated odorants with a single/identical analytical system, an analytical method should be developed to encompass all of the analytical procedures that have been developed for each odorant.

In this study, a GC-mass spectrometry (MS)-based analytical method has been developed to cover a maximum range of odorants with a single analytical system. This system is based on sorbent tube (ST) sampling with the aid of a thermal desorber (TD) technique. To this end, the maximum number of the designated odorants with diverse functionalities and related physicochemical properties were simultaneously collected in the same manner with a ST by considering all of the relevant quality assurance (QA) procedures. In addition, to obtain optimal recovery of each target odorant, appropriate TD conditions and MS methodologies were controlled and developed.

## Results and Discussion

### The maximum detectable range of target odorants with different sorbent tubes and thermal desorption conditions

In this study, the maximum range of target odorants that can be covered by a single analytical system was assessed by analyzing a total of 26 target odorants (consisting of liquid (n = 23) and gaseous working standards (n = 3)) using 3 types of ST combinations ((1) Carbopack C + B + X (CBX), (2) Tenax TA (Tenax), and (3) Carbopack C + X + Carboxen 1000 (CXCn)) at three different operation conditions (cold trap temperatures of −25, 0, and 25 °C). In Table S-1, the basic calibration data obtained for each individual odorant with each type of analytical conditions are summarized in terms of (1) the response factor (RF, ng^−1^), (2) the coefficient of determination (R^2^), and (3) the relative standard error (RSE, %).

#### EXP stage 1 results

In Exp stage 1, if the liquid working standard (L-WS) was analyzed by the CBX tube, the RF values of all of the target compounds (with the exception of propionaldehyde (PA), n-butyraldehyde (BA), and dimethyl sulfide (DMS)) were similar, regardless of the adsorption temperature of the cold trap (mean RSE values of the RF values (n = 20) obtained by three different cold trap temperatures = 1.57 ± 0.91%) (Figure S-1). However, the RF values of PA, BA, and DMS tended to decrease as the adsorption temperature of the cold trap increased. In the case of BA, the RF value of “CBX25 (sorbent tube code + adsorption temperature of cold trap in TD (Table 1))” was about 20% lower than those of “CBX-25” and “CBX0” (RF values: (1) 16,293 ng^−1^ (CBX-25), (2) 16,079 ng^−1^ (CBX 0), and (3) 13,553 ng^−1^ (CBX25)). In the case of PA and DMS, as the adsorption temperature of the cold trap increased from −25 to 0 °C, the RF values dropped dramatically by about 70% and 99%, respectively (changes in the RF values (CBX-25 to CBX0): (1) PA = 8,150 to 2,324 ng^−1^ and (2) DMS = 34,608 to 127 ng^−1^)). All target odorants, except DMS, had fairly good coefficients of determination (R^2^) of more than 0.99, regardless of the adsorption temperature of the cold trap. In the case of DMS, good linearity was seen only at “CBX-25”. The linearity of the DMS dropped dramatically to below 0.4 as the adsorption of the cold trap increased to 0 or 25 °C.

To learn more about this phenomenon, the adsorption capacity of the CBX tube was compared against the Tenax tube, which is one of the most widely used adsorbents for the collection of voltaic organic compound (VOC). To this end, the RF values of the 23 target VOCs contained in the L-WS were examined for the CBX and Tenax tube at a fixed adsorption temperature of the cold trap (i.e., −25 °C). The CBX tube had higher RF values for most VOCs (n = 18), except for the five carboxyl compounds, compared to the Tenax tube. In the case of light compounds, the RF values of the Tenax tube were noticeably lower than those of the CBX tube. For example, the RF value of DMS obtained by the CBX tube was about three times higher than that of the Tenax tube (RF value of DMS: (1) 34,608 ng^−1^ (CBX-25) and (2) 11,295 ng^−1^ (Tenax-25)). Other light aldehydes (n = 4) also had RF values that were about 120% higher for the CBX tube compared to the Tenax tube (Fig. 2).

#### EXP stage 2 results

In Exp stage 2, the gaseous working standard (G-WS) containing acetaldehyde (AA), hydrogen sulfide (H_2_S), and methanethiol (MT) was analyzed by using CBX and CXCn tubes with a fixed cold trap adsorption temperature (−25 °C). The Carboxen 1000, which was packed in the CXCn tube, has strong adsorptivity relative to CBX adsorbents. Although the adsorptivity of the CXCn tube is stronger than the CBX tube, H_2_S was not detected by either the CBX or CXCn tubes. Thus, H_2_S was excluded from the list of target compounds in this study. AA and MT were detected by the CBX and CXCn tubes; their RF values were below 40 ng^−1^. Considering that the mean RF value of the 23 target odorants obtained from Exp stage 1 is above 30,000 ng^−1^, the RF values of AA and MT obtained with the CBX and CXCn tubes were fairly low. However, the R^2^ values of AA and MT are significantly different between the CBX and CXCn tubes. The CBX tube had negative R^2^ values for AA (−12.9) and MT (−3.96). The calibration results for AA and MT obtained by the CBX tube are unreliable due to their negative R^2^ values. In contrast, the R^2^ values of AA and MT obtained by the CXCn tube were high (above 0.95). As a result, in the case of the CXCn tube analysis, reliable calibration data for AA and MT can be acquired with high R^2^ values despite their low RF values. A line of evidence indicates that MT can be broken down to other sulfides depending on the thermal desorption conditions (through catalytic reactions)^8^. The recovery of MT should be reduced if such breakdown is to occur. However, as all ST holders used in this study were made of quartz, catalytic reactions of MT were least likely to occur. In line with such expectation, the occurrence of other sulfides (i.e., trisulfides) was not detected from MS searching of our TD-based analysis (at 320 °C desorption).

### Comparison of odorant detectability between different studies

In this section, the detectability of our ST/TD-GC-MS system was assessed using both L-WSs and G-WSs. The detection limit of 25 target odorants (with the exception of H_2_S) was calculated as the method detection limit (MDL) by following the relevant U.S. EPA guidelines^9^. The MDL values were compared with the emission standard levels of the KMOE as a simple means to test the practicality of our proposed method. In other words, if the obtained MDL values are far higher than the ambient levels of odorants, the application of this approach should be limited to a narrow range. In addition, the MDL values were compared between our single analytical system and five different analytical systems for the analysis of different odorants (a complicated system)^4^.

The MDL values of 25 target odorants expressed initially in mass quantities were translated into air concentrations using ideal gas equation assuming a 1 L sample volume (ideal gas equation: PV = nRT; pressure (P) = 1 atm, sample volume (V) = 1 L, mole (n) = mass (g)/MW (g/mole), R = 0.082057 atm·L·mole^−1^·K^−1^, and temperature (T) = 298.15 K). Accordingly, 22 target odorants had fairly low MDL values with a mean of 51.9 ± 67.1 ppt (with the exception of DMS, AA, and MT). In the case of DMS, although a low MDL was recorded in “CBX-25” (with a value of 11.2 ppt), the MDL values of DMS increased to ppb levels as the adsorption temperature of the cold trap was increased ((MDL values: (1) CBX-25 = 11.2 ppt, (2) CBX0 = 3.07 ppb, and (3) CBX25 = 19.9 ppb). The MDL values of AA and MT were moderately higher than those of the other target odorants (MDL values: (1) CBX-25 = 54.8 ppb (AA) and 86.7 ppb (MT) and (2) CBX-25 = 63.7 ppb (AA) and 56.4 ppb (MT)) (Table 2).

The MDL values for the 25 target odorants obtained by our single analytical system (ST/TD-GC-MS) were similar to those obtained by a variety of recommended analytical systems (Table 3). For example, the MDL of the five designated odorant aldehydes (AA, PA, BA, isovaleraldehyde (IA), and n-Valeraldehyde (VA)) obtained with the 2,4-dinitrophenylhydrazine (DNPH) cartridge-high-performance liquid chromatography (HPLC)-UV system, which is one of the recommended methods for their analysis, generally ranged from 0.01 to 0.1 ppb levels^10^. In this study, although AA had a high MDL value (about 50 ppb), the MDL values of the other aldehydes (PA, BA, IA, and VA) were similar to the DNPH-HPLC-UV system and ranged from 0.02 to 0.11 ppb. In the case of TMA and carboxyl compounds, our single analytical system had MDL values lower than those of the recommended ones. The MDL value for TMA using our system was 100 times lower than the SPME-GC-MS system that is generally recommended for TMA analysis (MDL of TMA: (1) this study = 0.02 ppb and (2) recommended system = 2.38 ppb) (Table 3). Carboxyl compounds (propionic acid (PPA), n-butyric acid (BTA), isovaleric acid (IVA), and n-valeric acid (VLA)) also had lower MDL values with our ST/TD-GC-MS system than those of a previously used analytical system (SPME-GC-MS) (MDL of carboxyl compounds: (1) this study = 0.02 to 0.12 ppb and (2) recommended system = 1.32 to 1.80 ppb).

The detection limits for 17 of the designated odorants obtained by our ST/TD-GC-MS system were much lower than the regulation guidelines for the emission concentrations set by the KMOE (with the exception of ammonia, DMS, AA, and MT): 17 designated odorants - (1) mean MDL value by our study = 0.06 ± 0.08 ppb and (2) mean emission standard concentration by the KMOE = 1,550 ± 3,569 ppb (Table 2). In the case of DMS, a low MDL value can be acquired by using a −25 °C cold trap during adsorption sampling: DMS – (1) MDL value of “CBX-25” = 0.01 ppb, (2) MDL value of “Tenax-25” = 0.03 ppb, and (3) emission standard concentration = 10 ppb. However, the MDL values of AA and MT were higher than their emission standard levels (Table 2). As a result, 20 out of the 22 designated odorants (with the exception of ammonia and H_2_S) can be quantified meaningfully by the single ST/TD-GC-MS system. In addition, in the case of 18 quantifiable odorants, it is possible to detect their concentrations at significantly lower levels than the emission standard levels suggested by the KMOE (Figure S-2).

### Results of the environmental sample analysis

To validate the effectiveness of our single analytical system for the quantitation of maximum odorants from real environmental samples^11^, air samples obtained from an organic fertilizer plant were obtained and analyzed with our ST/TD-GC-MS system using the “CBX-25” approach. The ability to simultaneously analyze the designated odorants from the field samples was assessed in reference to their emission guidelines (Table 2). The three field samples from (1) the slurry treatment facility, (2) compost facility, and (3) ambient air in the organic fertilizer plant were collected with CBX tubes and analyzed.

23 out of the 25 target odorants (with the exception of AA and MT) were detected in the three field samples. In all three field samples, the concentrations of three carboxyl compounds (BTA, IVA, and VLA) were higher than the emission guideline levels determined by the KMOE ((1) slurry treatment facility: 1.74 (IVA) – 12.1 (BTA) ppb, (2) compost facility: 4.34 (IVA) – 34.3 (BTA) ppb, and (3) ambient air: 0.90 (IVA) – 4.75 (BTA) ppb) (Table S-2). The concentrations of DMS detected from the slurry treatment and compost facilities also exceeded the emission guideline levels with values of 18.2 and 12.2 ppb, respectively. TMA was only detected in the compost facility sample (6.86 ppb); its levels from the slurry treatment facility and ambient air samples were at MDL levels.

The results of the odorant analysis from the organic fertilizer plant samples were similar to those reported by previous studies. Jo, *et al*.^12^ quantified diverse malodors containing 22 of the designated odorants from a slurry treatment facility (liquid fertilizer system), compost facility, and ambient air (background air) in a swine facility located in Korea. Jo, *et al*.^12^ observed DMS in the concentration range between 3.31 ppb (slurry treatment facility) and 9.47 ppb (compost facility). In this study, the concentrations of DMS in analogous samples were determined to be in a comparable range between 18.2 ppb (slurry treatment facility) and 12.2 ppb (compost facility). The four aldehydes (PA, BA, IA, and VA) measured in this work were also comparable to the results of Jo, *et al*.^12^ (ranging from 1 to 3 ppb). In the case of carboxyl compounds (PPA, BTA, IVA, and VLA), Jo, *et al*.^12^ found lower concentrations: 0.12 ppb (VLA, slurry treatment facility) to 5.11 ppb (PPA, slurry treatment facility). In this study, their counterparts were determined to range from 1.74 ppb (VLA, slurry treatment facility) to 34.3 ppb (BTA, compost facility) (Table S-2). Blanes-Vidal, *et al*.^13^ also analyzed diverse odorants emitted from swine slurry samples. RSC and carboxyl compounds were detected predominantly in the swine slurry sample (concentrations of RSC and carboxyl compounds: (1) RSC (DMS and dimethyl disulfide (DMDS)) = 0.20 to 602 ppb and (2) carboxyl (PPA, isobutyric acid (IBA), BTA, IVA, VLA, n-hexanoic acid (HXA), and n-heptanoic acid (HPA)) = 0.09 to 256 ppb). The concentrations of aldehyde and hydrocarbon compounds in their swine slurry samples were recorded at sub-ppb levels.

## Conclusions

In order to carry out reliable quantitation of diverse malodors in air, the combined application of several types of analytical methods and systems is needed due to the complexities involved in the analysis. In this study, a single analytical system based on the ST/TD-GC-MS system was developed to simultaneously analyze a maximum number of odorants. In the course of this study, we attempted to determine the optimal conditions for an analytical system that can ensure the maximum recovery of odorants in reference to the procedure recommended by the KMOE (e.g., the determination of ST types and cold trap temperatures).

Based on our newly established procedure, we were able to reliable analyse up to 18 out of the 22 odorants designated by the KMOE (with the exception of ammonia, H_2_S, AA, and MT) using a single ST/TD-GC-MS system. The relatively high recovery of target odorants was recorded by a CBX tube sampler with the TD operated at −25 °C. The MDL values of 18 odorants (out of the 22 designated odorants) obtained by our analytical system were a fairly low, ranging from 0.01 ppb (DMDS) to 0.12 ppb (PPA). These MDL levels were significantly lower than the emission standard levels set by the KMOE (mean 1,473 ± 3,491 ppb, n = 18) as well as their threshold values (mean 350 ± 1,243 ppb, n = 18). Although our simplified analytical system allowed for quantitative analysis of AA and MT, their MDL values were moderately higher than the emission standard levels and threshold values (concentrations of AA and MT = (1) MDLs = 86.7 and 54.8 ppb, (2) emission standard levels = 50 and 2 ppb, and (3) threshold values = 6 to 145 ppb and 0.07 to 1.05 ppb, respectively). Because there are few limitations in the application of our ST/TD-GC-MS method, such a system can be applied for the simple analysis of diverse odorants and air pollutants with reliable QA data to replace complicated, multi-component analytical systems.

## Materials and Methods

### Experimental scheme

**Figure 1:**
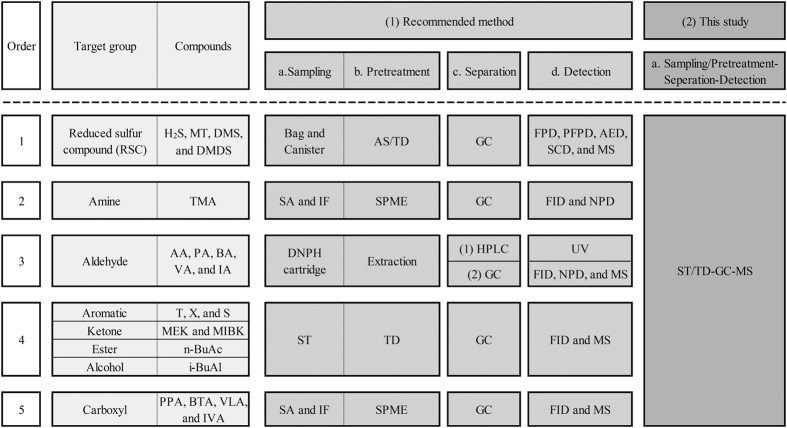
Comparison of the analytical systems for the analysis of offensive odorants: the recommended methods vs. the simplified approach used in this study. Abbreviations: [A] Sampling (SA = solvent absorption, IF = impregnated filter, DNPH = 2,4-dinitrophenylhydrazine, and ST = sorbent tube). [B] Pretreatment (AS = air server, TD = thermal desorber, and SPME = solid phase microextraction). [C] Separation (GC = gas chromatography and HPLC = high-performance liquid chromatography). [D] Detection (FPD = flame photometric detector, PFPD = pulsed FPD, AED = atomic emission detector, SCD = sulfur chemiluminescence, MS = mass spectrometry, FID = flame ionization detector, NPD = nitrogen phosphorus detector, and UV = ultraviolet).

**Figure 2:**
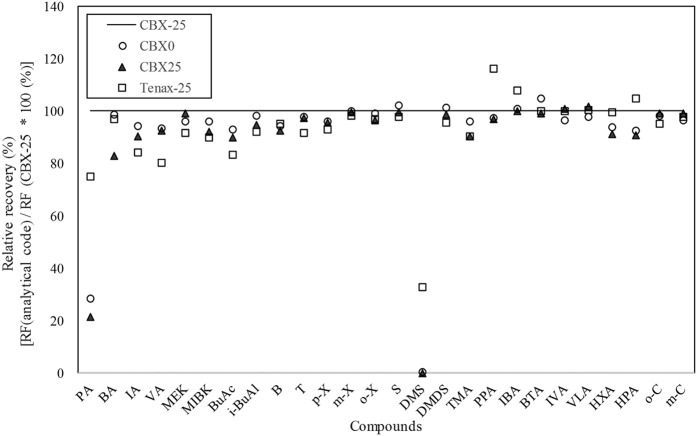
Relative recovery (%) of liquid VOC standards (n = 23) between different experimental stages.

In this research, an experimental method for the simultaneous analysis of diverse odorous compounds was developed using a single experimental setup consisting of an ST/TD-GC-MS system (Fig. 1). To this end, in our research, the sum of up to 21 offensive odor substances designated by the KMOE (except for ammonia) along with volatile fatty acids and cresols (with low odor thresholds) were initially selected as the target analytes (n = 26). The analysis and validation of these targets were carried out using three different types of experimental stages, as described in Table 1.

#### Exp stage 1

Calibration of the L-WSs: 23 odorants (18 odorants designated by the KMOE plus five VOCs) were prepared in liquid phase standards. The L-WSs, containing the 23 odorants, were loaded on the ST and analyzed using the TD-GC-MS system. The relative recovery of each target odorant was evaluated (1) across three different adsorption temperatures of the cold trap (−25, 0, and 25 °C) and (2) between two different ST types (CBX vs. Tenax TA).

#### Exp stage 2

Calibration of the G-WSs: For a number of volatile species (AA, H_2_S, and MT), G-WSs were purchased and used for calibration after completing the proper dilution. These were then analyzed by the ST/TD-GC-MS system with two different ST types ((1) CBX tube and (2) CXCn tube).

#### Exp stage 3

Monitoring of the environmental samples: The odorant samples collected from agricultural facilities (such as an organic fertilizer plant at a pig farm) were analyzed by combining the CBX tube with the TD-GC-MS system, as defined in Exp stages 1&2. Based on the analysis of various odorants from real samples, the effectiveness of the analytical method for diverse odorants was validated.

### Selection and preparation of working standards

In the beginning stage of this work, up to 26 compounds were selected as targets (Table S-3). (1) Here, 21 of them belong to a list of 22 odorants designated by the KMOE: AA, PA, BA, IA, VA, methyl ethyl ketone (MEK), methyl isobutyl ketone (MIBK), n-butyl acetate (BuAc), isobutyl alcohol (i-BuAl), toluene (T), xylenes (p, m, o-X), styrene (S), H_2_S, MT, DMS, TMA, PPA, BTA, IVA, and VLA. (2) In addition, five more compounds were added as references due to their low threshold values: benzene (B), IBA, HXA, HPA, and cresols (o, m-C).

Ammonia has a relatively low molecular weight of 17 g/mole and a high volatility. Thus, it is difficult to obtain a high recovery with an ST packed with Carbopack adsorbents. In addition, the main spectrum of ammonia (17 m/z) is very different from those of the other common target analytes (34 to 108 m/z) used in this study. For these reasons, ammonia was excluded from the list of target analytes. To assess the maximum covering range of odorants for quantitative analysis, a liquid working standard containing 23 target compounds was prepared (after excluding 3 compounds: AA, H_2_S, and MT). For these 3 compounds, gaseous standards were also used as-purchased (in cylinder, Rigas, Korea). Note that H_2_S and MT are in gaseous phase at room temperature. In the case of AA, if liquid phase standard is used, the recovery can be affected sensitivity by the types of solvent used^14^.

The reagent grade chemicals (RGCs) of each target analyte (with the exception of AA, H_2_S, and MT) were purchased at a purity ranging from 97.0 to 99.0% (Sigma-aldrich, USA). The primary standards (PSs) were prepared separately (PS-1, PS-2, and PS-3) by mixing the RGCs: (1) PS-1 (n = 12) consisted of aldehyde (n = 4), ketone (n = 2), ester (n = 1), alcohol (n = 1), and aromatic (n = 4); (2) PS-2 (n = 3) consisted of DMS, DMDS, and TMA; and (3) PS-3 (n = 8) consisted of carboxyl (n = 7) and cresol (n = 1). The mean concentrations of PS-1, PS-2, and PS-3 were 59.2 ± 3.12 μg μL^−1^, 120 ± 13.3 μg μL^−1^, and 106 ± 5.22 μg μL^−1^, respectively. The first liquid working standard (1st L-WS) was made by mixing the three PSs (PS-1 = 6 μL and PS-2 and PS-3 = 4 μL) with methanol (≥99.8%, Burdick & Jackson, USA) to make a 4 mL solution (mean ± SD concentration = 98.5 ± 12.9 ng μL^−1^). The final L-WS for the four-point calibrations was prepared by dilution of the 1st L-WS with methanol to generate four different concentration levels (mean ± SD concentration = 9.85 ± 1.29 ng μL^−1^ to 98.5 ± 12.9 ng μL^−1^) (Table S-4).

The PS gases of AA, H_2_S, and MT were purchased individually in two cylinders (Rigas Corp., Korea). The first cylinder was prepared such that it contained only AA at 99.6 ppm (certification data: 31 Dec. 2014 and expiration date: 30 Dec. 2015) and the second cylinder had 20.7 ppm of both H_2_S and MT (certification data: 26 Feb. 2015 and expiration date: 25 Aug. 2015) (experiment period: 1–31 May 2015). The G-WSs for the calibration of the three compounds were prepared by diluting PS gases in the cylinders with ultra-pure nitrogen (purity > 99.999%) in a 1 L polyester aluminum (PEA) bag: 204 ± 4.50 to 2,044 ± 45.0 ppb (Table S-4). In order to minimize the loss of the G-WS by the bag material, 1 L PEA bag was filled with the PS gases for surface coating (12 hours) before using for this experiment.

### Instrumental system

For the quantitative analysis of target odorants, we used a combination of an ST and a TD as a pretreatment tool. The ST was analyzed using a GC (Shimadzu GC-2010, Japan) equipped with an MS (Shimadzu QP2010 Ultra, Japan) and a TD (Markes International UNITY II, UK). The TD focusing trap was packed with Carbopack B and C (Markes International, UK) in a 1:1 volume ratio (ID: 2 mm, total sorbent bed length: 50 mm) (Table S-5).

To obtain the optimal recovery of all target odorants in the sampling and pretreatment stages, three different sampling tubes (two types of three-bed STs and a single bed (Tenax TA) tube) with different adsorptivities were prepared to allow for comparison between their recoveries: (1) CBX tube (Carbopack-C (70 mg), -B (50 mg), and -X (50 mg)), (2) CXCn tube (Carbopack-C (70 mg), -B (50 mg), and Carboxen 1000 (50 mg)), and (3) Tenax tube (Tenax TA (100 mg))^15^. It is well-known that the multi-bed ST can yield high recovery when analyzing multiple VOCs with varying carbon numbers^15^,^16^,^17^,^18^.

The odorants loaded on the ST were thermally desorbed at 320 °C (5 min) in a reverse flow of 100 mL min^−1^ of a helium carrier gas (purity > 99.999%). The desorbed analytes were swept into the cold trap in the stream of the carrier gas. To compare the recovery as a function of the adsorption temperature of the cold trap, the performance of the cold trap was tested at three different temperature settings: −25, 0, and 25 °C. The cold trap was then rapidly desorbed (320 °C for 10 min) in a reverse flow of the carrier gas to transfer (inject) the target analytes into the CP-wax GC capillary column (CP-wax column: diameter of 0.25 mm, length of 30 m, and thickness of 0.25 μm). The wax column was selected for the optimal separation of polar compounds (e.g., carboxyl and phenol) from non-polar compounds. The oven temperature was initially set at 40 °C (for 10 min), ramped at 5 °C min^−1^ to 220 °C, and held at this temperature for 10 min (total run time of 56 min).

The analytes separated by the GC system were detected by the MS system. The interface and ion source temperatures were set to 230 °C. Analytes were initially analyzed in the total ion chromatographic (TIC) mode over a mass range of 33 (or 35) to 600 m/z. The extracted ion chromatographic (EIC) mode was subsequently applied to minimize interference and maximize recovery by using the significant ions that were identified from the spectrum of each analyte (Table S-3)^19^. The detailed instrumental conditions are presented in Table S-5.

### Experimental approaches

#### Exp stage 1- L-WS

To calibrate the L-WS, the inlet and outlet of the ST were connected to a filter tube packed with Carbopack X (100 mg) and a vacuum pump interfaced with a mass flow controller (Sibata ΣMP-30, Japan), respectively. The L-WS was injected onto the ST via a temporary injection port pierced into the Teflon tubing; this was used to connect the inlet of the ST and the filter tube^17^. At the same time, filtrated air was constantly supplied through the filter tube to the ST (purge flow rate of 100 mL min^−1^ for 3 min). The L-WS loaded onto the ST was then analyzed using the TD-GC-MS system.

#### Exp stage 2-G-WS

The inlet of the ST was connected to a PEA bag filled with the G-WS via Teflon tubing. The outlet of the ST was then connected to a vacuum pump, and the transfer of the G-WS into the ST was initiated at a fixed flow rate of 100 mL min^−1^ for 1 min. The ST that was loaded with the G-WS was then thermally desorbed to derive calibration curves for each of the three analytes (AA, H_2_S, and MT).

#### Exp stage 3- Environmental sample (organic fertilizer plant)

The field samples collected from an organic fertilizer plant in Chungnam, South Korea were used as an indirect means to validate the reliability of our experimental system. These samples were collected from three sampling points ((1) slurry treatment facility, (2) compost facility, and (3) ambient (air surrounding the study area)) using the CBX tube and analyzed by the TD-GC-MS system. To analyze these samples, the outlet of the CBX tube was connected to a vacuum pump, and the air samples were drawn into the CBX tube using the vacuum pump at a fixed flow rate of 50 mL min^−1^ over three intervals (0.2, 1, and 10 min) (Table 4). Considering the breakthrough and detection limit of target odorants, the collection of samples was made at 3 different sample volumes (10, 50, and 500 mL). Detailed descriptions of our experimental scheme including standard preparation, instrumental system, and experimental procedure are provided in the Supplementary Information (SI).

## Additional Information

**How to cite this article**: Kim, Y.-H. and Kim, K.-H. A novel quantitation approach for maximizing detectable targets for offensive/volatile odorants with diverse functional groups by thermal desorption-gas chromatography-mass spectrometry. *Sci. Rep.* **6**, 29248; doi: 10.1038/srep29248 (2016).

## Supplementary Material

Supplementary Information

## Figures and Tables

**Table 1:** Basic experimental scheme used to develop an experimental system for the maximum detectable targets of the designated offensive odorants by the ST/TD-GC-MS system.

[A] Basic information of the three experimental stages set for different target species (stage 1 vs. 2) or for different sample types (stages 1/2 vs. 3)				
Order		Exp code	Contents	
1		Exp stage 1	Analysis of liquid working standards (containing 18 designated odor substances and five VOCs) with different TD adsorption conditions	
2		Exp stage 2	Analysis of gaseous working standards containing acetaldehyde (AA), hydrogen sulfide (H_2_S), and methanethiol (MT)	
3		Exp stage 3	Analysis of environmental samples from a pigpen	
**[B] Sample code information**				
**Order**	**Analytical code**	**Adsorbent of sampling tube**	**Adsorption temp. of cold trap (°C)**	
1	CBX-25	Carbopack C (70 mg) + Carbopack B (50 mg) + Carbopack X (50 mg)	-25	
2	CBX0	“	0	
3	CBX25	“	25	
4	Tenax-25	Tenax TA (100 mg)	-25	
5	CXCn-25	Carbopack C (70 mg) + Carbopack X (50 mg) + Carboxen 1000 (50 mg)	-25	

**Table 2:** Method detection limits (MDLs) of 25 target VOCs (plus H_2_S) obtained by the TD-GC-MS technique.

Order	Compound	a. Method detection limit^a^ (MDL)										b. Emission Standard^c^ (ppb)	c. Threshold^d^
		*(a) Mass (ng)*					*(a) Concentration*^b^*(ppb)*						
		CBX-25	CBX0	CBX25	Tenax-25	CXCn-25	CBX-25	CBX0	CBX25	Tenax-25	CXCn-25		
**[A] Liquid working standards (L-WSs)**													
1	PA	0.27	0.96	1.28	0.36	–	0.11	0.40	0.54	0.15	–	50	1–8.7
2	BA	0.24	0.25	0.29	0.25	–	0.08	0.08	0.10	0.08	–	29	0.67–8.91
3	IA	0.07	0.07	0.08	0.08	–	0.02	0.02	0.02	0.02	–	3	0.1–2.24
4	VA	0.15	0.16	0.16	0.19	–	0.04	0.05	0.05	0.05	–	9	0.41–2.3
5	MEK	0.14	0.14	0.14	0.15	–	0.05	0.05	0.05	0.05	–	13,000	440–7760
6	MIBK	0.08	0.08	0.08	0.09	–	0.02	0.02	0.02	0.02	–	1,000	170–537
7	BuAc	0.22	0.24	0.25	0.27	–	0.05	0.05	0.05	0.06	–	1,000	16–195
8	i-BuAl	0.13	0.14	0.14	0.14	–	0.04	0.04	0.05	0.05	–	900	11–832
9	B	0.19	0.20	0.21	0.20	–	0.06	0.06	0.07	0.06	–	–	2700–3630
10	T	0.18	0.18	0.18	0.20	–	0.05	0.05	0.05	0.05	–	10,000	330–1550
11	p-X	0.12	0.12	0.13	0.13	–	0.03	0.03	0.03	0.03	–	1,000	58–490
12a	m-X	0.17	0.17	0.17	0.17	–	0.04	0.04	0.04	0.04	–	1,000	41
12b	o-X	0.16	0.16	0.16	0.16	–	0.04	0.04	0.04	0.04	–	1,000	380–851
12c	S	0.17	0.17	0.17	0.18	–	0.04	0.04	0.04	0.04	–	400	35
13	DMS	0.03	7.79	50.4	0.09	–	0.01	3.07	19.9	0.03	–	10	2.24–3
14	DMDS	0.04	0.04	0.04	0.04	–	0.01	0.01	0.01	0.01	–	9	2.2–12.3
15	TMA	0.06	0.06	0.06	0.06	–	0.02	0.02	0.03	0.03	–	20	0.032–2.4
16	PPA	0.37	0.38	0.39	0.32	–	0.12	0.13	0.13	0.11	–	30	6–145
17	IBA	0.30	0.30	0.30	0.28	–	0.08	0.08	0.08	0.08	–	–	1.5–19.5
18	BTA	0.06	0.06	0.06	0.06	–	0.02	0.02	0.02	0.02	–	1	0.19–3.89
19	IVA	0.17	0.18	0.17	0.17	–	0.04	0.04	0.04	0.04	–	1	0.078–2.46
20	VLA	0.12	0.12	0.12	0.12	–	0.03	0.03	0.03	0.03	–	0.9	0.037–4.79
21	HXA	0.13	0.14	0.14	0.13	–	0.03	0.03	0.03	0.03	–	–	0.6–12.6
22	HPA	0.12	0.13	0.13	0.11	–	0.02	0.02	0.02	0.02	–	–	27.5
23a	o-C	0.10	0.10	0.10	0.10	–	0.02	0.02	0.02	0.02	–	–	0.054–1.7
23b	m-C	0.08	0.08	0.08	0.08	–	0.02	0.02	0.02	0.02	–	–	0.1–0.794
**[B] Gaseous working standards (G-WSs)**													
24	AA	156	–	–	–	115	86.7	–	–	–	63.7	50	6–145
25	H_2_S	NA	–	–	–	NA	NA	–	–	–	NA	20	0.41–17.8
26	MT	108	–	–	–	111	54.8	–	–	–	56.4	2	0.07–1.05

^a^The product of the standard deviation of seven replicates multiplied by the Student’s t-value at the 99.9% confidence level (6 df, t = 3.14).

^b^Pressure (P) = 1 atm, sample volume (V) = 1 L, R = 0.082057 atm·L·mole^−1^·K^−1^, and temperature (T) = 298.15 K. Concentration (ppb) = (mass (ng)/sample volume (L)) × (R (atm·L·mole^−1^·K^−1^) × T (K)/P (atm))/MW (g/mole).

^c^The emission standard levels were determined by the Korea Ministry of Environment (KMOE^3^).

^d^Nagata & Takeuchi^2^ and Schiffman *et al*.^20^.

**Table 3:** Comparison of detection limits for the designated offensive odor substances obtained by different analytical systems between previous studies and this study.

Order	Target group	Analytical system^a^	Detection limits (ppb)		Target compounds	Reference
			MDL	LOD^b^		
**[A] Previous studies**						
1a	RSC	AS/TD-GC-PFPD	0.016–0.049	–	H_2_S, MT, DMS, and DMDS	Susaya, *et al*.^21^
1b		AS/TD-GC-PFPD	0.02–0.03	–	H_2_S, MT, DMS, and DMDS	Jo, *et al*.^22^
1c		AS/TD-GC-PFPD	–	0.05–0.07	H_2_S, MT, DMS, DMDS, COS, and CS_2_	Trabue, *et al*.^23^
1d		AS/TD-GC MS	–	0.07–0.01		
2	Amine	SPME-GC-MS	2.38	–	TMA	Kim, *et al*.^24^
3a	Aldehyde	DNPH-HPLC-UV	0.02–0.04		AA, PA, BA, IA, and VA	Sandner, *et al*.^10^
3b		DNPH-HPLC-UV	–	0.08–0.10	AA, PA, BA, IA, and VA	Pal & Kim^25^
4a	Aromatic, ketone, ester, and alcohol	ST/TD-GC-MS	0.004–0.018^c^	–	T, p-,m-,o-X, S, MEK, MIBK, BuAc, and i-BuAl	Kim & Kim^26^
4b		ST/TD-GC-MS	0.032–0.140^c^	0.001–0.005^c^	T, p-,m-,o-X, S, MEK, MIBK, BuAc, and i-BuAl	Kim & Kim^19^
5	Carboxyl	SPME-GC-MS	1.32–1.80	–	PPA, BTA, IVA, and VLA	Kim, *et al*.^24^
**[B] This study**^c^						
1	RSC	ST/TD-GC-MS	0.010–0.011	0.001–0.002	DMS and DMDS	
2	Amine		0.02	0.002	TMA	
3	Aldehyde		0.02–0.11	0.002–0.009	PA, BA, IA, and VA	
4	Aromatic, ketone, ester, and alcohol		0.02–0.06	0.001–0.002	T, p-,m-,o-X, S, MEK, MIBK, BuAc, and i-BuAl	
5	Carboxyl		0.02–0.12	0.002–0.013	PPA, BTA, IVA, and VLA	

^a^Refer to the Fig. 1.

^b^Calculated using three times the standard deviation of the background noise.

^c^The mass MDL values are translated into air concentration MDL values assuming a sample volume of 1 L.

**Table 4:** Information about the analytical approach used at each of all three experimental stages.

[A] Analysis of working standards in both liquid (Exp stage 1) and gas phases (Exp stage 2)							
Order			Exp stage 1: L-WS analysis		Exp stage 2: G-WS analysis		
1	Standard phase:		Liquid		Gas		
2	Target compounds:		18 designated odor substances and five VOCs		AA, H_2_S, and MT		
3	Calibration range:		4.92 ± 0.64 ng to 49.2 ± 6.43 ng		35.2 ± 5.97 ng to 352 ± 59.7 ng		
4	Calibration approach^a^		Direct injection (fixed standard volume approach)		Direct loading (fixed standard volume approach)		
5	Analytical volume^b^		0.5 μL		100 mL		
6	Analytical code:		CBX-25, CBX0, CBX25, and Tenax-25		CBX-25 and CXCn-25		
7	Purge (or G-WS) flow rate:		200 mL min^−1^		50 mL min^−1^		
8	Purge (or G-WS) loading time:		1.5 min		2 min		
9	Purge (or G-WS) loading volume:		300 mL		100 mL		
**[B] Analysis of environmental samples (Exp stage 3)**							
Order	Sampling location	Environmental sample code	Sampling point	Analytical code	Sampling flow rate (mL min^−1^)	Sampling volume (mL)	Temp. (°C)
1	Organic	A	Slurry treatment facility	CBX-25	50	10 and 500	25
2	Fertilizer plant	B	Compost facility	“	50	50 and 500	25
3	(Nonsan, Chungnam, Korea)	C	Ambient	“	50	500	25

^a^Kim & Kim^17^.

^b^Volume of the working standard used for TD-based calibration.
