# Supplementary material for: A novel quantitation approach for maximizing detectable targets for offensive/volatile odorants with diverse functional groups by thermal desorption-gas chromatography-mass spectrometry
Source: Sci Rep. 2016 Jul 11;6:29248. doi: 10.1038/srep29248 (PMC4941403; doi:10.1038/srep29248)
Supplement: Supplementary Information [file srep29248-s1.pdf]

Supporting Information

A novel quantitation approach for maximizing detectable targets for offensive/volatile odorants with diverse functional groups by thermal desorption-gas chromatography-mass spectrometry

Yong-Hyun Kim, Ki-Hyun Kim*
Department of Civil and Environmental Engineering, Hanyang University,
222 Wangsimni-Ro, Seoul 04763, Korea

* Corresponding author: kkim61@hanyang.ac.kr or kkim61@nate.com, Phone: 82-2-2220-2325. Fax -1945

References

1. KMOE. Annual Environmental Report in South Korea. Korean Ministry of Environment (2008) Available at: http://webbook.me.go.kr/DLi-File/091/023/002/186050.pdf (Accessed: 15 Mar 2016)
2. Nagata, Y. & Takeuchi, N. Measurement of odor threshold by triangle odor bag method. Odor Measurement Review, Office of Odor, Noise and Vibration Environmental Management Bureau, Ministry of the Environment, Tokyo, Japan. 118-127 (2003).
3. Schiffman, S. S., Bennett, J. L., Raymer, J. H. Quantification of odors and odorants from swine operations in North Carolina. *Agr. Forest Meteorol.* **108**, 213-240 (2001).

Supplementary figure legends

Figure S1 | Chromatograms of the liquid VOC standards for different experimental stages (concentration = 19.7 ± 2.57 ng μL^{-1} and analytical volume = 0.5 μL). [A] Amine (n=1), aldehyde (n=4), ketone (n=2), ester (n=1), and alcohol (n=1). [B] Aromatics (n=4). [C] Sulfide (n=2). [D] Carboxyl (n=7) and phenol (n=1).

Figure S2 | Comparison of the relative sensitivity of 23 VOCs: detection limit vs. emission standard (KMOE[1]) or odor threshold (Nagata & Takeuchi[2] and Schiffman, et al.[3]).

Table S-1 | Comparison of the basic calibration results between different sampling tubes and TD conditions: (1) response factor (RF), (2) coefficient of determination (R^2), and (3) relative standard error (RSE).

Order	Compound	a. Response factor (RF, ng^{-1})					b. Coefficient of determination (R^2)					c. Relative standard error (RSE, %)				
		CBX-25	CBX0	CBX25	Tenax-25	CXCn-25	CBX-25	CBX0	CBX25	Tenax-25	CXCn-25	CBX-25	CBX0	CBX25	Tenax-25	CXCn-25
**[A] Exp stage 1: Liquid working standards (L-WSs)**																
1	PA	8,150	2,324	1,742	6,117	-	0.9988	0.9964	0.9900	0.9920	-	0.51	0.68	0.51	0.99	-
2	BA	16,293	16,079	13,553	15,816	-	0.9968	0.9955	0.9970	0.9955	-	0.73	1.21	2.25	2.33	-
3	IA	28,925	27,277	26,218	24,446	-	0.9981	0.9954	0.9929	0.9970	-	3.50	1.21	2.14	1.59	-
4	VA	28,256	26,490	26,223	22,790	-	0.9984	0.9966	0.9982	0.9912	-	0.49	0.90	1.20	2.66	-
5	MEK	25,131	24,145	24,927	23,105	-	0.9958	0.9935	0.9958	0.9938	-	0.75	0.81	1.55	2.73	-
6	MIBK	35,969	34,645	33,167	32,407	-	0.9974	0.9943	0.9940	0.9965	-	1.18	1.20	0.89	0.39	-
7	BuAc	30,577	28,446	27,537	25,541	-	0.9987	0.9960	0.9943	0.9957	-	0.54	2.51	0.74	1.65	-
8	i-BuAl	28,822	28,350	27,369	26,651	-	0.9977	0.9929	0.9932	0.9994	-	0.89	3.10	4.01	3.01	-
9	B	23,272	21,992	21,616	22,144	-	0.9932	0.9903	0.9929	0.9908	-	0.91	1.15	2.12	2.87	-
10	T	53,659	52,651	52,301	49,354	-	0.9914	0.9950	0.9994	0.9976	-	1.21	1.56	2.34	2.18	-
11	p-X	57,951	55,875	55,475	53,882	-	0.9979	0.9949	0.9963	0.9929	-	0.86	2.01	1.54	1.91	-
12a	m-X	57,264	57,451	57,049	56,390	-	0.9948	0.9950	0.9939	0.9941	-	0.59	0.88	1.18	1.55	-
12b	o-X	60,399	59,999	58,380	58,730	-	0.9961	0.9944	0.9940	0.9955	-	1.01	0.95	1.32	0.58	-
12c	S	50,434	51,619	50,378	49,501	-	0.9938	0.9942	0.9945	0.9971	-	1.11	0.87	3.05	0.64	-
13	DMS	34,608	127	20	11,295	-	0.9992	0.3987	-1,376	0.9446	-	0.41	0.88	2.01	3.65	-
14	DMDS	44,031	44,606	43,533	42,191	-	0.9949	0.9903	0.9933	0.9906	-	0.52	0.34	0.58	1.34	-
15	TMA	39,533	38,039	35,784	35,753	-	0.9973	0.9932	0.9943	0.9966	-	0.98	1.12	1.10	1.67	-
16	PPA	4,332	4,226	4,197	5,044	-	0.9997	0.9930	0.9912	0.9992	-	3.40	3.01	1.45	2.74	-
17	IBA	3,910	3,957	3,913	4,232	-	0.9995	0.9904	0.9919	0.9944	-	0.89	2.43	1.01	2.31	-
18	BTA	15,054	15,801	14,973	15,073	-	0.9976	0.9995	0.9937	0.9991	-	4.02	4.10	3.02	1.99	-
19	IVA	14,166	13,701	14,329	14,160	-	0.9977	0.9978	0.9961	0.9997	-	1.26	2.25	1.23	2.34	-
20	VLA	19,423	18,993	19,780	19,509	-	0.9933	0.9997	0.9978	0.9994	-	2.95	2.10	2.01	1.49	-
21	HXA	19,689	18,520	18,038	19,613	-	0.9977	0.9917	0.9957	0.9984	-	2.69	3.22	2.22	2.31	-
22	HPA	17,514	16,254	15,904	18,364	-	0.9968	0.9996	0.9991	0.9976	-	0.82	1.55	3.51	1.56	-
23a	o-C	29,816	29,389	29,606	28,370	-	0.9943	0.9977	0.9910	0.9909	-	0.87	1.64	2.51	1.19	-
23b	m-C	37,051	35,798	36,809	36,320	-	0.9908	0.9961	0.9951	0.9937	-	1.91	0.89	3.68	0.78	-
**[B] Exp stage 2: Gaseous working standards (G-WSs)**																
24	AA	25	-	-	-	34	-12.86	-	-	-	0.9686	26.3	-	-	-	10.9
25	H_2S	ND[a]	-	-	-	ND	ND	-	-	-	ND	ND	-	-	-	ND
26	MT	36	-	-	-	35	-3.955	-	-	-	0.9662	20.5	-	-	-	11.6

[a]Not detected

Table S-2 | Validation of a single sampling/analysis technique for odorants: Concentrations of target odorants determined from three types of environmental samples[a].

Order	Compound	a. Concentration (ppb)			b. Emission standard[b] (ppb)
	Air-sampling point:	Slurry treatment facility	Compost facility	Ambient	
1	PA	2.78	1.55	3.59	**50**
2	BA	0.08	1.94	0.28	**29**
3	IA	0.02	3.07	0.81	**3**
4	VA	0.04	0.04	1.06	**9**
5	MEK	522	79.5	1.31	**13,000**
6	MIBK	5.37	0.76	0.04	**1,000**
7	BuAc	0.08	0.09	0.21	**1,000**
8	i-BuAl	9.25	6.22	0.09	**900**
9	B	17.7	0.54	0.42	**-**
10	T	12.3	4.15	6.08	**10,000**
11	p-X	0.40	0.30	0.22	**1,000**
12a	m-X	0.87	0.63	0.45	**1,000**
12b	o-X	0.47	0.32	0.22	**1,000**
12c	S	0.76	0.80	0.33	**400**
13	DMS	**18.2**	**12.2**	0.05	**10**
14	DMDS	1.81	1.42	0.03	**9**
15	TMA	0.03	6.86	0.03	**20**
16	PPA	11.30	29.3	7.58	**30**
17	IBA	10.78	38.6	1.86	**-**
18	BTA	**12.1**	**34.3**	**4.75**	**1**
19	IVA	**8.81**	**33.5**	**1.54**	**1**
20	VLA	**1.74**	**4.34**	**0.90**	**0.9**
21	HXA	1.05	1.89	0.14	**-**
22	HPA	0.19	0.14	0.02	**-**
23a	o-C	0.05	0.08	0.02	**-**
23b	m-C	0.25	0.08	0.02	**-**

[a]All environmental samples were analyzed using the "CBX-25 approach"

[b]The emission standard levels were determined by the Korea Ministry of Environment (KMOE[1])

Below detection limit

Exceeding the emission standard

0

Table S-3 | List of 21 designated odor substances and five reference VOCs selected for the development of the ST/TD-GC-MS technique.

Order	Group	Full name	Short name	MW (g/mol)	Density (g mL^{-1})	Formula	CAS number	Main spectra[a] (m/z)
**[A] Liquid working standards (L-WSs)**								
1	*Aldehyde*	Propionaldehyde	PA	58.1	0.798	C_3H_6O	123-38-6	58
2		n-Butyraldehyde	BA	72.11	0.805	C_4H_8O	123-72-8	41, 42, 43, 72
3		Isovaleraldehyde	IA	86.1323	0.797	$C_5H_{10}O$	590-86-3	41, 43, 44
4		n-Valeraldehyde	VA	86.1323	0.81	$C_5H_{10}O$	110-62-3	41, 44
5	*Ketone*	Methyl ethyl ketone	MEK	72.11	0.805	C_4H_8O	78-93-3	43, 72
6		Methyl isobutyl ketone	MIBK	100.2	0.802	$C_6H_{12}O$	108-10-1	41, 43
7	*Ester*	n-Butyl acetate	BuAc	116.16	0.881	$C_6H_{12}O_2$	123-86-4	41, 43
8	*Alcohol*	Isobutyl alcohol	i-BuAl	74.12	0.801	$C_4H_{10}O$	78-83-1	41, 42, 43
9	*Aromatic*	Benzene	B	78.11	0.878	C_6H_6	71-43-2	78
10		Toluene	T	92.14	0.866	C_7H_8	108-88-3	91, 92
11		p-Xylene	p-X	106.16	0.865	C_8H_{10}	106-42-3	91, 105, 106
12a		m-Xylene	m-X	106.16	0.865	C_8H_{10}	108-38-3	91, 105, 106
12b		o-Xylene	o-X	106.16	0.88	C_8H_{10}	95-47-6	91, 105, 106
12c		Styrene	S	104.15	0.909	C_8H_8	100-42-5	78, 103, 104
13	*Sulfide*	Dimethyl sulfide	DMS	62.134	0.846	C_2H_6S	75-18-3	62
14		Dimethyl disulfide	DMDS	94.199	1.046	$C_2H_6S_2$	624-92-0	94
15	*Amine*	Trimethylamine	TMA	59.11	0.67	C_3H_9N	75-50-3	58, 59
16	*Carboxyl*	Propionic acid	PPA	74.0785	0.99	$C_3H_6O_2$	79-09-4	73, 74
17		Isobutyric acid	IBA	88.1051	0.9697	$C_4H_8O_2$	79-31-2	73
18		n-Butyric acid	BTA	88.1051	0.96	$C_4H_8O_2$	107-92-6	60, 73
19		Isovaleric acid	IVA	102.1317	0.93	$C_5H_{10}O_2$	503-74-2	60
20		n-Valeric acid	VLA	102.1317	0.94	$C_5H_{10}O_2$	109-52-4	60, 73
21		n-Hexanoic acid	HXA	116.1583	0.927	$C_6H_{12}O_2$	142-62-1	60, 73
22		n-Heptanoic acid	HPA	130.1849	0.9181	$C_7H_{14}O_2$	111-14-8	60, 73
23a	*Phenol*	o-Cresol	o-C	108.1378	1.0465	C_7H_8O	95-48-7	107, 108
23b		m-Cresol	m-C	108.1378	1.034	C_7H_8O	108-39-4	107, 108
**[B] Gaseous working standards (G-WSs)**								
1	*Aldehyde*	Acetaldehyde	AA	44.05	0.784	C_2H_4O	75-07-0	44
2	*Sulfide*	Hydrogen sulfide	H_2S	34.08	1.363	H_2S	7783-06-04	34
3		Methanethiol	MT	48.11	0.9	CH_4S	74-93-1	48

[a]Mass spectra selected for the EIC-based analysis

(Grey shading) Designated offensive odor substances determined by the Korea Ministry of Environment (KMOE[1]); 21 out of the 22 designated odorants were selected in this study (with the exception of ammonia)

Table S-4 | Basic information regarding the preparation of liquid and gaseous phase standards of the VOC.

[A] Exp stage 1: Liquid working standards (L-WSs)

a. Preparation of three types of primary standards (PS-1, PS-2, and PS-3): Mixing of reagent grade chemicals (RGC) in three separate (2 mL) vials

Order	Compounds	**RGC** (%)	**PS[a]-1** (ng μL^{-1})	Compounds	**RGC** (%)	**PS[a]-2** (ng μL^{-1})	Compounds	**RGC** (%)	**PS[a]-3** (ng μL^{-1})
(a) Group 1: Aldehyde, ketone, ester, alcohol, and aromatic				(b) Group 2: Sulfide and amine			(c) Group 3: Carboxyl and phenol		
1	PA	97.0	55,290	DMS	99.0	104,693	PPA	99.0	108,900
2	BA	99.0	56,925	DMDS	99.0	129,443	IBA	99.0	106,667
3	IA	97.0	55,221	TMA	25.0	125,625	BTA	99.0	105,380
4	VA	97.0	56,121				IVA	99.0	101,750
5	MEK	99.0	56,925				VLA	99.0	103,180
6	MIBK	99.5	56,999				HXA	99.0	101,970
7	BuAc	99.5	62,614				HPA	99.0	100,991
8	i-BuAl	99.0	56,642				o-C	99.0	115,115
9	B	99.5	62,401				m-C	99.0	113,740
10	T	99.5	61,548						
11	p-X	99.0	61,168						
12	m-X	99.0	61,168						
13	o-X	97.0	60,971						
14	S	99.0	64,279						
Mixing recipe in volume (μL)[b]:		RGC	each 140		RGC of DMS and DMDS	each 250		RGC	each 200
		Total	1,960		RGC of TMA	1500		Total	1,800
					Total	2,000			

Table S-4 | Continued.

b. Liquid working standards (L-WSs): Mixing of PS-1, PS-2, PS-3, and methanol

Order	Compounds		1st L-WS[c] (ng μL^{-1})		Final L-WS (ng μL^{-1}) for four-point calibration analysis: 1st	2nd	3rd	4th
1	PA		82.9		8.29	16.6	41.5	82.9
2	BA		85.4		8.54	17.1	42.7	85.4
3	IA		82.8		8.28	16.6	41.4	82.8
4	VA		84.2		8.42	16.8	42.1	84.2
5	MEK		85.4		8.54	17.1	42.7	85.4
6	MIBK		85.5		8.55	17.1	42.7	85.5
7	BuAc		93.9		9.39	18.8	47.0	93.9
8	i-BuAl		85.0		8.50	17.0	42.5	85.0
9	B		93.6		9.36	18.7	46.8	93.6
10	T		92.3		9.23	18.5	46.2	92.3
11	p-X		91.8		9.18	18.4	45.9	91.8
12	m-X		91.8		9.18	18.4	45.9	91.8
13	o-X		91.5		9.15	18.3	45.7	91.5
14	S		96.4		9.64	19.3	48.2	96.4
15	DMS		105		10.5	20.9	52.3	105
16	DMDS		129		12.9	25.9	64.7	129
17	TMA		126		12.6	25.1	62.8	126
18	PPA		109		10.9	21.8	54.5	109
19	IBA		107		10.7	21.3	53.3	107
20	BTA		105		10.5	21.1	52.7	105
21	IVA		102		10.2	20.4	50.9	102
22	VLA		103		10.3	20.6	51.6	103
23	HXA		102		10.2	20.4	51.0	102
24	HPA		101		10.1	20.2	50.5	101
25	o-C		115		11.5	23.0	57.6	115
26	m-C		114		11.4	22.7	56.9	114
Mixing recipe in volume (μL)[b]:		PS-1	6					
		PS-2	4	1st L-WS	200	400	1,000	2,000
		PS-3	4					
		MeOH	3,986	MeOH	1,800	1,600	1,000	0
		Total	4,000	Total	2,000	2,000	2,000	2,000

[a]Primary standard (PS) used to make the liquid-phase working standard
[b]Mixing conditions and formula for PS and L-WS are given
[c]The first liquid working standard (1st L-WS): Mixing of PS-1, PS-2, PS-3, and methanol

[B] Exp stage 2: Gaseous working standards (G-WSs)

Order	*a. Primary gas standards*: Cylinder[a]	Compounds	Concentration (ppm)	*b. Gaseous working standards* (a) Mixing volume (mL): 1st point	2nd point	3rd point	4th point	(b) Concentration (ppb): 1st point	2nd point	3rd point	4th point
1	A	AA	99.6	2	5	10	20	199	498	996	1,992
2	B	H_2S	20.7	10	25	50	100	207	518	1,035	2,070
3		MT	20.7	10	25	50	100	207	518	1,035	2,070
4	N_2	Nitrogen	> 99.999%	978	945	890	780				

[a]Rigas Corp., Korea

Table S-5 | Operational conditions for the analysis of 26 target compounds by the TD-GC-MS system.

**A. Thermal desorber (model: UNITYII, Markes, UK)**			
a. Sampling tube			
1. Trap tube:	Quartz (length: 90 mm, OD: 6.4 mm, and ID: 4.2 mm)		
2. Adsorbent:	(a) CBX, (b) Tenax, and (c) CXCn		
3. Desorption flow:	100 mL min^{-1} (to cold-trap)		
4. Desorption time:	5 min		
5. Desorption temp.:	320 ℃		
b.Cold-trap			
1. Trap tube:	Quartz (length: 100 mm, OD: 3.2 mm, and ID: 2 mm)		
2. Adsorbent:	Carbopack C and B (volume ratio = 1:1)		
3. Adsorption temp.:	(a) -25 ℃, (b) 0 ℃, and (c) 25 ℃		
4. Desorption temp.:	320 ℃ (to GC)		
5. Desorption flow:	14 mL min^{-1}		
c. Carrier gas setting			
1. Carrier gas:	Helium (> 99.999%)		
2. Constant gas pressure:	25 psi		
3. Initial gas flow rate:	2 mL min^{-1}		
3. Split flow:	10 mL min^{-1}		
4. Purge gas flow:	2 mL min^{-1}		
d. Line and interface temp.: 200 ℃			
**B. Gas chromatography (model: GC-2010, Shimadzu, Japan)**			
a. Column:	CP-wax (Agilent J&W, USA)<br>(length: 60 m, diameter: 0.25 mm, and film thickness: 0.25 µm)		
b. Oven setting:	40 ℃ (10 min) → 5 ℃/min → 220 ℃ (10 min)<br>(Total program time = 56 min)		
**C. Mass spectrometry (model: GCMS-QP2010 Ultra, Shimadzu, Japan)**			
a. Ionization mode:	EI (70 eV)	d. TIC scan range:	33~600 m/z
b. Ion source temp.:	230 ℃	e. Scan speed:	1250
c. Interface temp.:	230 ℃		

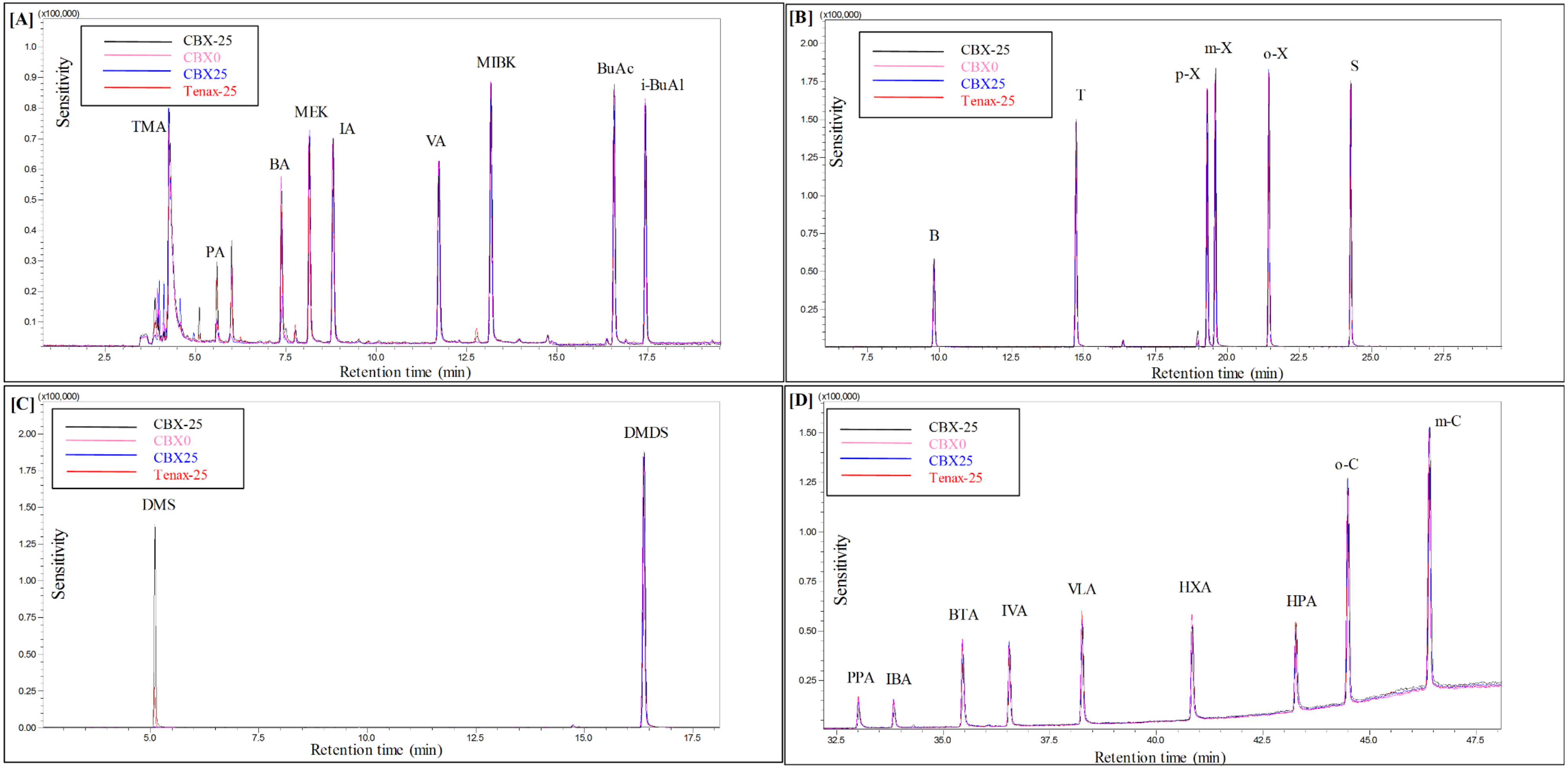

Figure S-1 | Chromatograms of the liquid VOC standards for different experimental stages (concentration = 19.7 ± 2.57 ng μL^{-1} and analytical volume = 0.5 μL).

[A] Amine (n=1), aldehyde (n=4), ketone (n=2), ester (n=1), and alcohol (n=1). [B] Aromatics (n=4). [C] Sulfide (n=2). [D] Carboxyl (n=7) and phenol (n=1).

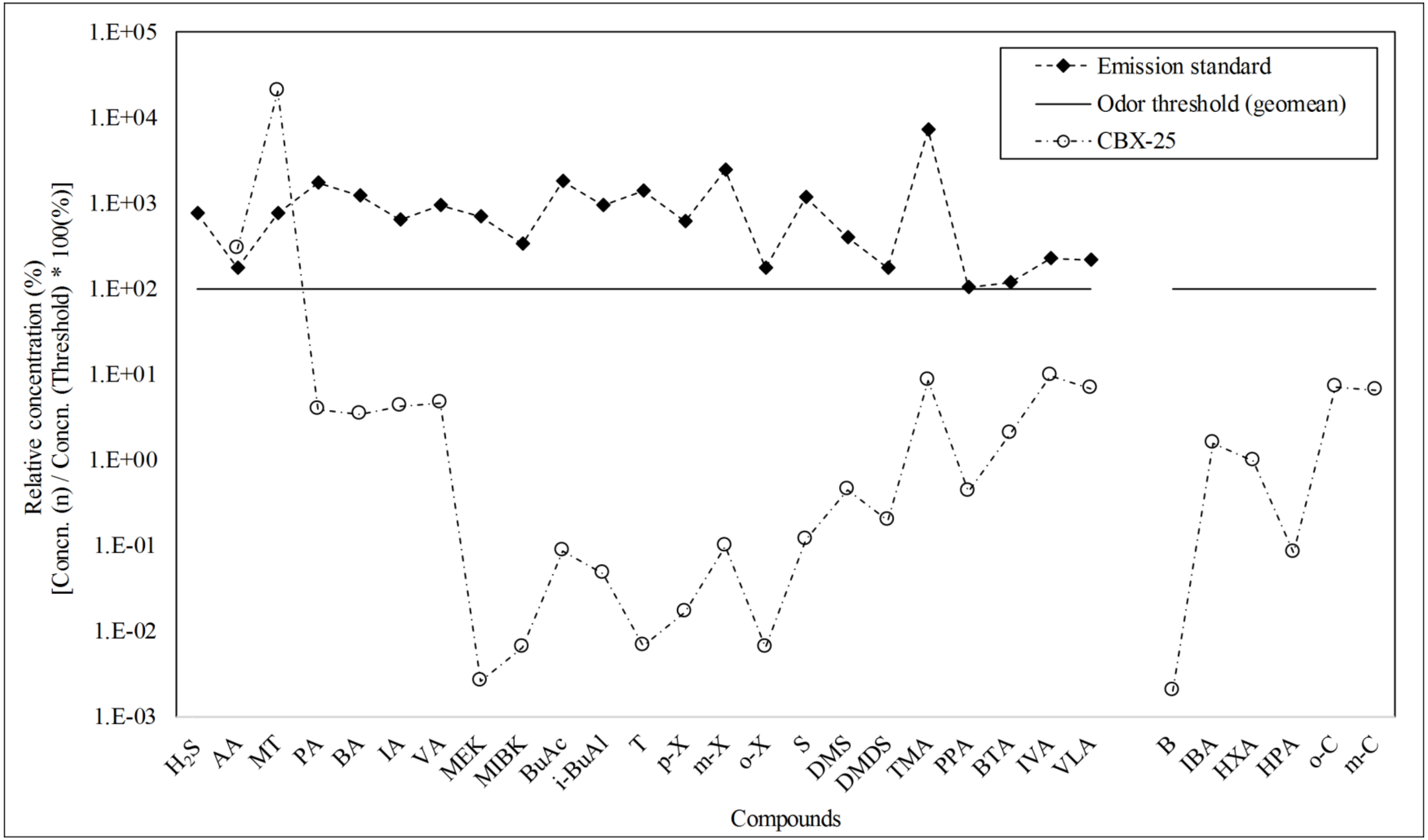

Figure S-2 | Comparison of the relative sensitivity of 23 VOCs: detection limit vs. emission standard (KMOE[1]) or odor threshold (Nagata & Takeuchi[2] and Schiffman, et al.[3]).
